# Induction of DNMT1-dependent demethylation of SHP-1 by the natural flavonoid compound Baicalein overcame Imatinib-resistance in CML CD34^+^ cells

**DOI:** 10.1186/s12964-023-01049-9

**Published:** 2023-03-03

**Authors:** Xuefen Xu, Shufan Ji, Yuan Chen, Siwei Xia, Yang Li, Li Chen, Yujia Li, Feng Zhang, Zili Zhang, Shizhong Zheng

**Affiliations:** 1 Department of Pharmacology, School of Medicine and Holistic Integrative Medicine, Nanjing University of Chinese Medicine, No.138, Xianlin Road, Nanjing, Jiangsu People’s Republic of China; 2 Jangsu Key Laboratory for Pharmacology and Safety Evaluation of Chinese Materia Medica, School of Pharmacy, Nanjing University of Chinese Medicine, Nanjing, People’s Republic of China

**Keywords:** CML CD34^+^ cells, DNMT1, SHP-1, Resistance, Baicalein

## Abstract

**Background:**

The most significant cause of treatment failure in chronic myeloid leukemia (CML) is a persistent population of minimal residual cells. Emerging evidences showed that methylation of SHP-1 contributed to Imatinib (IM) resistance. Baicalein was reported to have an effect on reversal of chemotherapeutic agents resistance. However, the molecular mechanism of Baicalein on JAK2/STAT5 signaling inhibition against drug resistance in bone marrow (BM) microenvironment that had not been clearly revealed.

**Methods:**

We co-cultured hBMSCs and CML CD34^+^ cells as a model of SFM-DR. Further researches were performed to clarify the reverse mechanisms of Baicalein on SFM-DR model and engraftment model. The apoptosis, cytotoxicity, proliferation, GM-CSF secretion, JAK2/STAT5 activity, the expression of SHP-1 and DNMT1 were analyzed. To validate the role of SHP-1 on the reversal effect of Baicalein, the SHP-1 gene was over-expressed by pCMV6-entry shp-1 and silenced by SHP-1 shRNA, respectively. Meanwhile, the DNMT1 inhibitor decitabine was used. The methylation extent of SHP-1 was evaluated using MSP and BSP. The molecular docking was replenished to further explore the binding possibility of Baicalein and DNMT1.

**Results:**

BCR/ABL-independent activation of JAK2/STAT5 signaling was involved in IM resistance in CML CD34^+^ subpopulation. Baicalein significantly reversed BM microenvironment-induced IM resistance not through reducing GM-CSF secretion, but interfering DNMT1 expression and activity. Baicalein induced DNMT1-mediated demethylation of the SHP-1 promoter region, and subsequently activated SHP-1 re-expression, which resulted in an inhibition of JAK2/STAT5 signaling in resistant CML CD34^+^ cells. Molecular docking model indicated that DNMT1 and Baicalein had binding pockets in 3D structures, which further supported Baicalein might be a small-molecule inhibitor targeting DNMT1.

**Conclusions:**

The mechanism of Baicalein on improving the sensitivity of CD34^+^ cells to IM might be correlated with SHP-1 demethylation by inhibition of DNMT1 expression. These findings suggested that Baicalein could be a promising candidate by targeting DNMT1 to eradicate minimal residual disease in CML patients.

**Video Abstract**

**Supplementary Information:**

The online version contains supplementary material available at 10.1186/s12964-023-01049-9.

## Background

The BCR-ABL TK inhibitors (TKIs) IM was identified as the most effective inhibition for TK activity in treatment of CML [1]. However, in most CML patients, IM does not eliminate minimal residual disease, which results in disease recurrence at discontinuation of IM treatment [2]. The persistence of CML subpopulation was critical for reason of molecular relapse after TKI cessation [3]. Recently, increasing articles were mainly concentrated upon BCR-ABL kinase-independent mechanisms, which might be involved in TKI resistance [4–6]. BM microenvironment is rich of various cytokines, which may provide CML cells persistence and prevent from TKIs-induced apoptosis [7]. Supplement of the supernatant isolated from BM stromal, could attenuate inhibition of CML CD34^+^ cells proliferation and colony forming ability [8]. The emerging evidence revealed that microenvironmental crosstalks might prevent CML CD34^+^ subpopulation from TKI-mediated apoptosis [9, 10]. BM microenvironment-mediated resistance has been paid much attention. And there was an urgent requirement to find novel strategies for reversing resistance.

DNA methylation was a critical epigenetic modification, which played important roles in regulation of constitutive survival pathway. Hypomethylated DNA was closely associated with tumor formation and development [11]. The DNA methyltransferase DNMT1 was involved in regulation of DNA methylation to silence tumour suppressor genes [12]. DNMT1 modified DNA methylation along with the PTPN6 promoter replication [13]. The Src homology region 2 domain-containing phosphatase-1(SHP-1) is a negative regulator of intracellular signaling [14]. The SHP-1 methylation led to gene silencing, which played an essential role in the formation of leukaemia resistance [15, 16]. In lymphoma and leukaemia cells, there was complete absence or partial reduction of SHP-1 expression at protein level. The degree of SHP-1 methylation in patients might be related with CML progression in various phases. SHP-1 promoter methylation was reduced following enhancement of SHP-1 expression in CML-CP patients [17].

SHP-1 could induce inhibition of STAT5 pathway [18] and dephosphorylation of JAK kinases [19]. Some target anticancer-drugs such as dovitinib were known to trigger cells growth inhibition and apoptosis through inducing the phosphatase activity of SHP-1 in carcinoma treatment [20]. Either JAK2 or BCR-ABL led to the activation of STAT5, which could drive cell cycle progression and upregulate survival genes expression [21]. However, comparing to normal hemopoiesis, the role of JAK2/STAT5 signaling activation in BCR-ABL^+^ leukemias was still paradoxical. Investigations from dominant negative JAK2 mutant [22] and BCR-ABL transduced JAK2 knockout murine BM cells [23] indicated that STAT5 was directly phosphorylated by BCR-ABL. We speculated that, on the base of inhibition of the BCR-ABL activity, JAK2/STAT5 pathway in pharmacologic interference appeared a promising therapeutic strategy in resistant CML.

A major bioactive flavones, Baicalein (5,6,7-trihydroxyfavone), is separated from the root of Scutellaria baicalensis Georgi. Nowadays, due to low toxicity and general safety of Baicalein,, our interests were aroused. Furthermore, the reversed effects of Baicalein on resistance were verified on many kinds of carcinoma cells. Baicalein increased the inhibitory effect of cisplatin on A549 lung adenocarcinoma cells through PI3K/Akt/NF-κB signaling [24]. In addition, Baicalein enhanced hepatocellular carcinoma cells sensitivity to chemotherapy by inducing apoptosis and attenuating P-glycoprotein activity [25]. Chen et al. reported Baicalein triggered mitochondria relevant apoptosis and enhanced the anticancer therapy of vincristine on CCRF-CEM leukemic cells [26]. Further revelation of the molecular mechanism of Baicalein against drug resistance in CML was urgently required before tracking it as clinical implications in treatment. In the current study, we found Baicalein had a reverse effect on IM resistance of CML CD34^+^ cells both in SFM-DR and engraftment model. The effects of Baicalein on reversing resistance could be association with demethylation of the SHP-1 promoter region in CML.

## Methods

### Subjects

Blood samples were collected from CML patients at chronic phase in Zhongda Hospital Southeast University, Nanjing, China. Leukopheresis samples were underway for CD34^+^ cells separation with CliniMACS (Miltenyi Biotech, Germany). CD34^+^ cells were selected by using anti-CD34 magnetic beads in a magnetic activated cell sorter system (Miltenyi Biotec). All subjects signed an informed consent form. The procedure of cell collection from patients conformed to guidelines in the Declaration of Helsinki, and was approved by the Institutional Review Board of Zhongda Hospital Southeast University.

### Cell culture

Human CML K562 cells and Human bonemesenchymal stem cells (hBMSCs) were acquired from the American Type Culture Collection (ATCC). hBMSCs were maintained in DMEM/F12 including 10% fetal bovine serum. K562 CD34^+^ cells were isolated by using anti-CD34 magnetic beads. CD34^+^ cells were incubated with or without hBMSCs at 37 °C with 5% CO_2_ in high GF-supplemented(stem cell factor 100 pg/mL, SCF + FL + TPO, 50 ng/ml) serum-free expansion medium(Stem Cell Technologies, Vancouver, BC, Canada). Baicalein and IM were purchased from Melonepharma (Dalian, China). Anti-human GM-CSF (α-rhGM-CSF) was acquired from PeproTech Inc.(USA).

### Apoptosis, proliferation ratio, Inhibitory ratio and colony-forming cell assays

K562 CD34 + cells and primary CD34 + CML cells were cultured with or without hBMSCs for 12 h in 6-well plates at a density of 2.5 × 10^5^ cells/well. Then the CD34 + cells were treated with various concentrations of IM (0, 0.125, 0.25, 0.5 μM) or Baicalein (0, 5, 10, 20 μM) for 36 h, respectively. The treated cells were measured by DAPI and the Annexin V/PI Cell Apoptosis Detection Kit (KeyGen Biotech, Nanjing, China) according to the manufacturer’s recommendations. For inhibitory ratio, the CD34^+^ cells (10^5^ cells/well in 96-well plates) were treated with IM or Baicalein at different doses with or without supernatant of hBMSCs for 36 h. Then, the proliferation ratio of CD34^+^ cells was performed by MTT assay. Inhibitory ratio(%) = (1-proliferation ratio) × 100%. For colony forming capacity assays, CD34^+^ cells (2.5 × 10^5^ cells/well in 6-well plates) were incubated with Baicalein (20 μM) and/or IM(0.5 μM) for 36 h in or not in hBMSCs and then transferred to 6-well plates by incubation at 37 °C for 28 days until visualization.

### Electrophoretic mobility shift assays (EMSA)

CML CD34^+^ cells (2 × 10^6^ cells/well in 75cm^2^ culture plates) were incubated with Baicalein (0, 5, and 20 μM) for 36 h in BM microenvironment. Nuclear extract was prepared as previously mentioned and EMSA was carried out according to the manufacturer’s description(Beyotime Institute of Biotechnology, Shanghai, China). The STAT5 oligonucleotide probe labeled with biotin was utilized to assess the specificity of protein binding to DNA. This assay was described as previously [27].

### Western blot analysis

K562 CD34^+^ cells and primary CML CD34^+^ cells(2.5 × 10^5^ cells/well in 6-well plates) were exposed to various stimulating conditions for the indicated times. The lysated preparations were carried out by Western blot as previously mentioned. Antibodies for Western blot were used in our research: p-JAK2^Tyr1007/1008^, p-STAT5^Tyr694^, JAK2, STAT5, BCR-ABL, CrkL, Survivin, Bcl-2, Mcl-1, XIAP, cleaved-caspase 3, bax, caspase 3, GAPDH, DNMT1 were obtained from Cell Signaling Technology (Danvers, MA, USA). SHP-1 and cyclin D2 were ordered from Abcam(Cambridge, MA, USA).

### DAPI staining, cell line transfection

DAPI staining was used to evaluate morphological features of apoptosis in cell nuclei as previously reported. The lentiviral vectors, psiHIV-mU6-shSHP-1 and psiHIV-mU6-empty control were constructed by GenePharma Co, Ltd, Shanghai, China. pCMV6-Entry shp-1 and pCMV6-Entry vector were provided by Sangon Biotechnology, Shanghai, China. Before transfection, ogarithmic growth K562 CD34^+^ cells were washed in antibiotic and serum-free medium, then seeded in 6-well plates with 5 × 10^4^/well cells. The ratio of lentiviral transfection system to medium containing 20% fetal bovine serum was 1:1. After transfection, all lentiviral knock-downs and plasmid were selected by incubating with puromycin(1 g/mL) for 72 h. Finally, the efficiency of transfection was examined using inverted fluorescence microscope.

### GM-CSF ELISA

GM-CSF concentration of supernatant was collected in the presence or absence of hBMSCs, and analyzed using human GM-CSF ELISA Kit from Beyotime Institute of Biotechnology (Shanghai, China) in accordance with the manufacturer’s instructions.

### Immunofluorescence confocal microscopy

CD34^+^ cells were tiled coverslips after various treatment (2.5 × 10^5^ cells/well in 6-well plates). And then cells in incubation with anti-p-STAT5^Tyr694^ antibody were processed for immunofluorescence staining as previously described [28].

### Cytokine antibody array

The supernatant of K562 CD34^+^ cells in hBMSCs or not was measured by the abcam Human Cytokine Antibody Array C Series 80 Targets (abcam, New England) according to the manufacturer’s instructions.

### Engrafted with human CD34^+^ cells in immunodeficient mice

K562 CD34^+^ cells (1 × 10^6^ cells/mouse) were collected, and inoculated via tail vein injection into 6–9 week-old NOD/SCID mice, which was accepted sublethal irradiation (300 cGy). Two days later, the mice transplanted with CD34^+^ cells were randomized into 4 groups respectively(5 mice per group): (1)DMSO group as a negative control; (2) Baicalein administration alone(20 mg/kg); (3) IM administration alone (200 mg/kg); (4) Baicalein combined with IM. The mice were given an intraperitoneal(i.p.) injection with or without Baicalein (20 mg/kg) every other day. IM(200 mg/kg) was given orally once every day. The transplanted mice were euthanized after 6 weeks and bone marrow cells, blood cells and spleen cells were harvested. To evaluate leukocyte proportion of human cell engraftment, cells signed with antihuman CD45-PE antibody(eBioscience) were analyzed through flow cytometry. Human specific cell subpopulations were measured by marking with antibodies to human CD34-FITC (eBioscience), CD33-FITC (eBioscience), and CD19-FITC(eBioscience). Human CD45^+^ cells from BM of mice were harvested via immunomagnetic activated cell selection. To assess BCR-ABL expressing cells in malignant engraftment, selected CD45^+^ cells from BM were assessed for BCR-ABL mRNA expression by qRT-PCR.

### The activity of DNMT1

Nuclear extract was carried out according to the manufacturer’s instruction (Beyotime Institute of Biotechnology, Shanghai, China). The activity of DNMT1 was detected by human DNA methyltransferase 1 (DNMT1) ELISA kit according to the manufacturer’s instructions(Epigentek, USA). The concentration of DNMT1 in samples were calculated by the working standard curve.

### qRT-PCR

Total RNA extracts were obtained by the previously mentioned method. The primers(Sangon Biotech (Shanghai) Co., Shanghai) were used in the reaction as follows:

BCR-ABL: forward, 5′-GGGCTCTATGGGTTTCTGAATG-3′ and reverse, 5′-CGCTGAAGGGCTTTTGAACT-3′;

GAPDH: forward, 5′-ACCCAGAAGACTGTGGATGG-3′, and reverse,

5′-TCTAGACGGCAGGTCAGGTC-3′.

### Methylation-specific PCR (MSP)

The SHP-1 DNA was amplified by qRT-PCR, using methylated or unmethylated CpG sequence as promoter-specific primer. The PCR reaction products were performed by agarose gel electrophoresis with a Tanon DNA marker(Tanon technology co., ltd, Shanghai, China) and 10,000X GelRed™ Nucleic Acid Gel Stain (Biotium, Inc., Hayward, CA, USA). The following SHP-1 gene primers for MSP PCR were used. M-MSP: Forward, 5′-GAACGTTATTATAGTATAGCGTTC-3'; reverse, 5′-TCACGCATACGAACCCA AACG-3'.

U-MSP: Forward, 5′-GTGAATGTTATTATAGTATAGTGTTTGG-3′; reverse,5′-TTCACACATACAAACCCA AACAAT-3′.

### Bisulfite sequencing PCR (BSP)

After treatment with bisulfite, unmethylated cytosines were completely converted to uracils, while methylated cytosines were still unchanged in Genomic DNA. The primers were designed according to the region of the CpG island for PCR. Then the purified PCR amplification was performed for TA cloning, and positive clones were used for sequencing. Finally, the measured sequences were compared with the original sequences, and the methylation degree was analyzed.

SHP-1-BSP: Forward, TATAGGGTTGTGGTGAGAAATTAATTAG; reverse, CATATATACCTTACACACTCCAAACCC.

### Statistical analysis

All data in the text were performed in triplicate and presented as the mean ± SD in a parallel manner. Statistical analyses were carried out by the GraphPad Prism software (GraphPad Software Inc., Avenida, CA, USA).

## Results

### BM microenvironment possessed anti-apoptotic effects on CML CD34^+^ cells

**Fig. 1:**
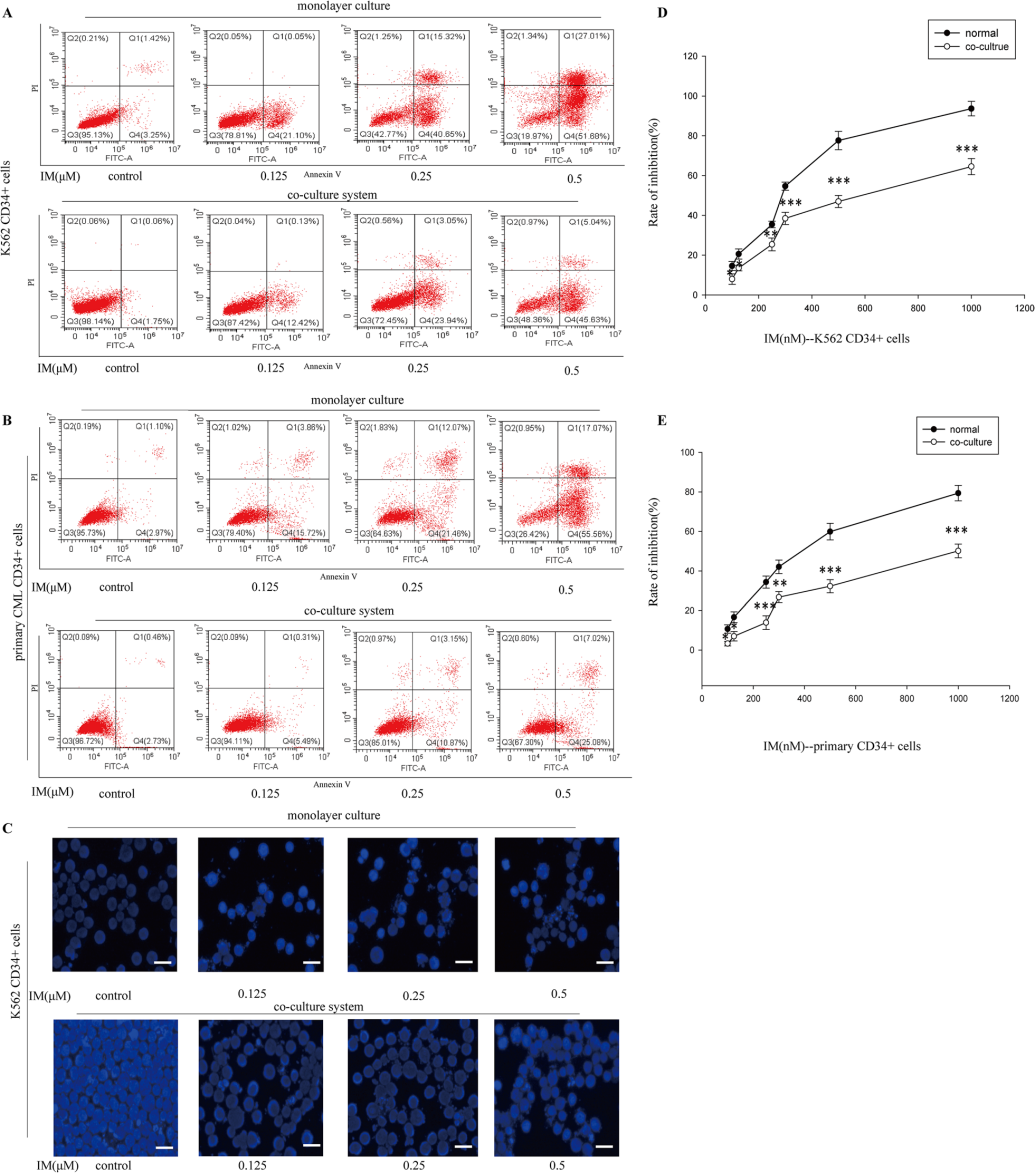
BM microenvironment protected CML CD34^+^ cells from IM-induced apoptosis. K562 CD34^+^ cells and primary CD34^+^ CML cells were cultured with or without hBMSCs for 12 h and then treated with various concentrations of IM (0, 0.125, 0.25, 0.5 μM) for 36 h, respectively. **A**, **B** Apoptosis was measured by Annexin V-PI double staining assay after treatment with IM in co-culture model or monolayer culture. **C** Apoptotic cells were observed by DAPI staining. Scale bar, 20 μm. **D** The growth inhibition effect of IM on CD34^+^ subpopulation in K562 cells or primary CML cells with or without the supernatant of hBMSCs was detected by MTT, and the inhibition rate (%) was evaluated. Data were expressed as means ± SD from three independent experiments. p* < 0.05, p** < 0.01, p*** < 0.001 versus the group treated with IM in monolayer culture

To imitate SFM-DR on CML CD34^+^ cells, we established co-culture model by using hBMSCs to mimic BM microenvironment [29]. AnnexinV/PI assays were applied. After administration with IM(0, 0.125, 0.25, 0.5 μM) for 36 h, the apoptosis rate of K562 CD34^+^ cells or primary CML CD34 + cells was markedly reduced in co-culture than in monolayer culture (Fig. 1A, B). Similar reduction was also observed by DAPI staining on morphologic alterations of the nucleus and apoptotic bodies in co-culture model (Fig. 1C). To observe the effect of culture supernatant (CM) of hBMSCs on cell viability, MTT was applied. As shown in Fig. 1D, E, CM of hBMSCs significantly reduced IM-induced inhibition in cell viability. Together, these data suggested that BM microenvironment potently induced CML CD34^+^ cells resistance, leading to the failure of clinically-available BCR-ABL TKIs in eradicating minimal residual disease.

### JAK2/STAT5 axis contributed to CML CD34^+^ cells resistance toward IM in BM microenvironment

**Fig. 2:**
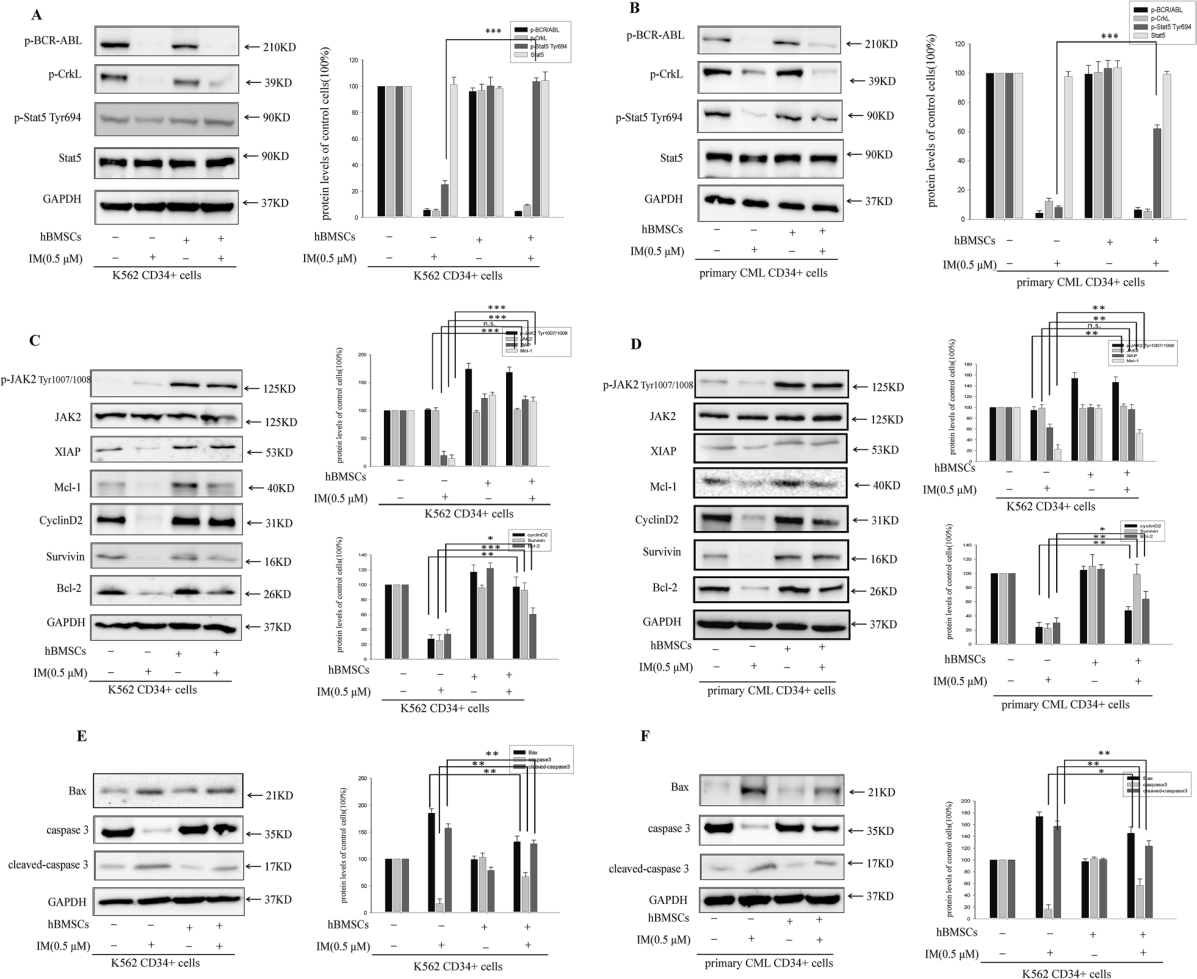
JAK2/STAT5 signaling and downstream protein were observed in CML CD34^+^ cells in BM microenvironment. BM microenvironment caused a BCR/ABL-independent activation of JAK2/STAT5 in the presence of IM in CML CD34^+^ cells. K562 CD34^+^ cells and primary CD34^+^ CML cells were cultured with or without hBMSCs for 12 h and then treated with various concentrations of IM (0, 0.125, 0.25, 0.5 μM) for 36 h, respectively. **A**, **B** Western blotting showed that activated BCR/ABL kinase was presented by staining for the expression of p-BCR/ABL and p-CrkL. STAT5 activation was shown by p-STAT5^Tyr694^-specific antibodies. GAPDH was regarded as loading control. The percentages of proteins p-BCR/ABL, p-CrkL and p-STAT5^Tyr694^ were observed by Western blot analysis in CML CD34^+^ cells. **C**, **D** The expressions of p-JAK2 ^Tyr1007/1008^, JAK2, XIAP, Mcl-1, CyclinD2, Survivin and Bcl-2 were determined using Western blot in CML CD34^+^ cells, respectively. **E**, **F** After IM treatment, the expression of pro-apoptotic proteins caspase3, cleaved-caspase 3 and Bax were determined by Western blot in K562 CD34^+^ cells and primary CD34^+^ CML cells. *p < 0.05, **p < 0.01, ***p < 0.001 the group treated with IM in monolayer culture versus the group treated with IM in co-culture system

To reveal the role of signal transduction in microenvironment induced-resistance, the phosphorylated status of STAT5 was assessed. Neither co-culture nor monolayer culture blocked BCR-ABL inactivation after treatment with IM (Fig. 2A, B). In fact, both BCR-ABL and CrkL were completely dephosphorylated by IM, indicating that BM microenvironment-mediated resistance was BCR-ABL independent. On the contrary, IM-induced STAT5 dephosphorylation of CD34^+^ cells in co-culture was reversed (Fig. 2A, B). These observations implicated an involvement of BCR-ABL-independent activation of STAT5 in BM microenvironment-induced CML CD34^+^ cells resistance to IM. So, it is worthy to investigate the further mechanism. As revealed in Fig. 2C, D, the significant increases of JAK2 phosphorylation were observed in BM Microenvironment, but JAK2 was not dephosphorylated by IM. Therefore, in co-culturing CML CD34^+^ cells, hBMSCs-mediated JAK2/STAT5 signaling was activated regardless of the presence of IM. Furthermore, as indicated in Fig. 2C, D, IM significantly reduced the expression of STAT5-target genes(Mcl-1 and XIAP) and anti-apoptotic proteins (Survivin, cyclin D2 and Bcl-2) in monolayer culture, but not in co-culture. Meanwhile, cleaved-caspase 3 and bax were significantly reduced by IM in co-culture comparing with monolayer culture (Fig. 2E, F). Thus, we considered that, CML CD34^+^ cells were reliant on BCR-ABL/STAT5 signaling for survival in monolayer culture, but became more dependent on JAK2/STAT5 signaling when BCR-ABL was fully inhibited by IM in co-culture.

### GM-CSF/DNMT1 was causal for activation of JAK2/STAT5 signaling in BM microenvironment-mediated resistance

**Fig. 3:**
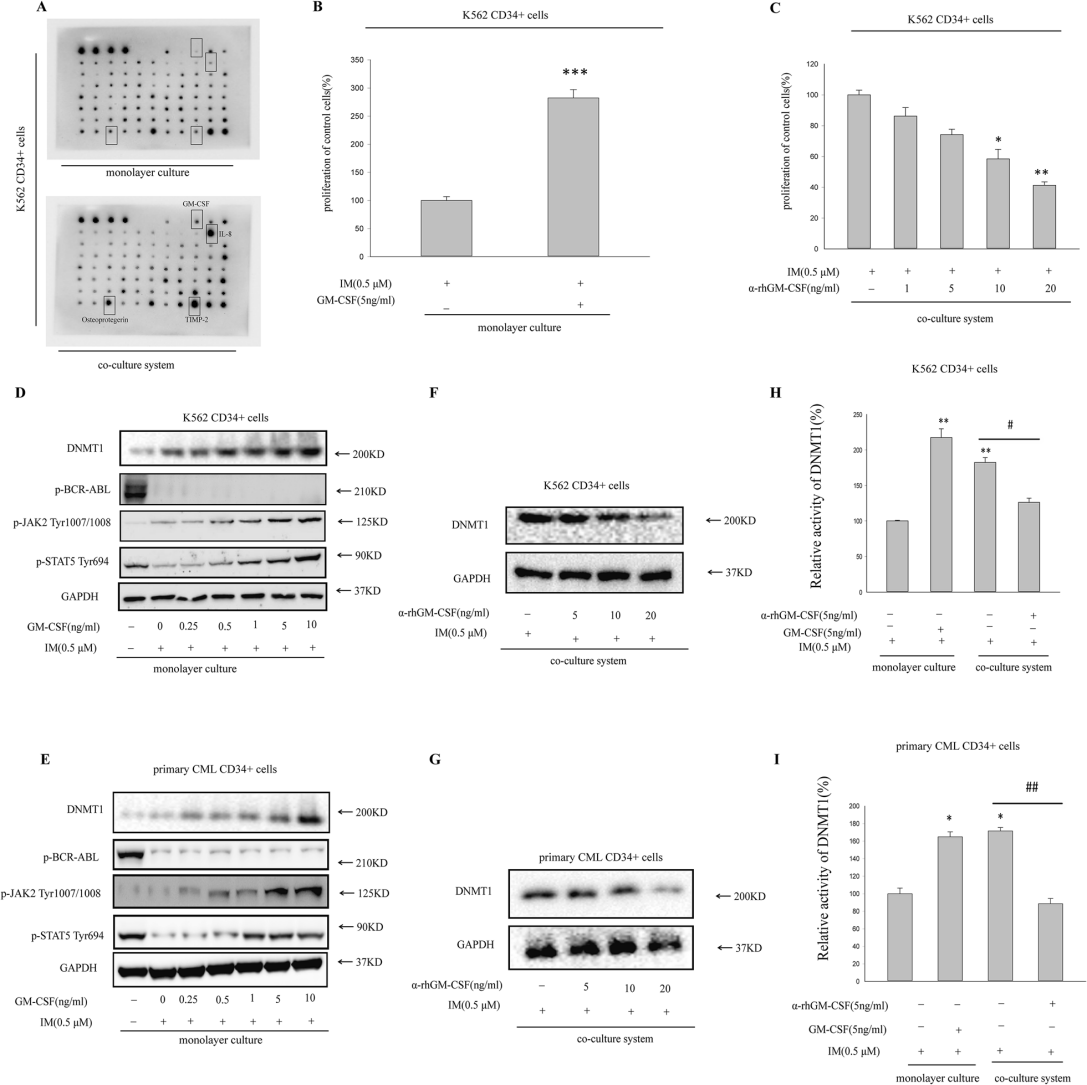
GM-CSF/DNMT1 mediated activation of JAK2/STAT5 signaling contributed to resistance in BM microenvironment. **A** Cytokine array. Different cytokines (80) were screened for differential expression in monolayer culture (up membrane) versus co-culture model in K562 CD34^+^ cells (low membrane). Different cytokines are spotted in duplicate on each membrane. Darker spots indicate higher expression. Positive controls (4 dots) are shown in the top left corners. Cytokines with increased expression are indicated by bold-lined squares in co-culture system versus thin-lined squares in monolayer culture. **B** The survival rate of K562 CD34^+^ cells after treatment with IM for 36 h in monolayer culture and GM-CSF was evaluated comparing with the control treatment (no GM-CSF) appling MTT assay. ***p < 0.001 versus control group. Data points of triplicate experiments are depicted. **C** Reversal of microenvironment-mediated IM resistance of K562 CD34^+^ cells by addition of increasing concentrations of neutralizing anti–human GM-CSF antibodies as indicated. *p < 0.05, **p < 0.01 versus control group. Data points of 3 independent experiments are depicted. **D**, **E** K562 CD34^+^ cells or primary CD34^+^ CML cells were treated with IM and the raise of GM-CSF dose as indicated in BM microenvironment or in monolayer culture. After treatment for 24 h, cell lysates were acquired for western blot with the indicated antibodies. GAPDH was regarded as loading control. **F**, **G** Supplementation of anti-human GM-CSF antibodies in co-culture system, DNMT1 expression was examined in K562 CD34^+^ cells or primary CD34^+^ CML cells. **H**, **I** K562 CD34^+^ cells or primary CD34^+^ CML cells were treated with IM, then GM-CSF or α- rhGM-CSF antibodies was added into BM microenvironment or monolayer culture, respectively. The activity of DNMT1 was detected by ELISA. *p < 0.05, **p < 0.01 versus CML CD34^+^ cells treated with IM in monolayer culture. ^#^*p* < 0.05, ^##^*p* < 0.01 versus CML CD34^+^ cells treated with IM in co-culture system

We hypothesized that cytokines might mediate BCR-ABL-independent activation of STAT5 in microenvironment. 80 targets of cytokines were detected by cytokine array. The concentrations of GM-CSF, IL-8, TIMP-2, and Osteopontin were obviously increased in co-culture compared with monolayer culture(Fig. 3A). However, only in GM-CSF-added monolayer medium, K562 CD34^+^ cells continued to grow slowly in the presence of IM(Fig. 3B), intimating that GM-CSF could induce an acquired IM resistance. As shown in Fig. 3C, supplementing anti-rhGM-CSF antibodies, which target GM-CSF, did not exhibit microenvironment-mediated protection in co-culture model. Moreover, GM-CSF-supplemented CML CD34^+^ cells could dose-dependently recover BCR-ABL-independent JAK2/STAT5 phosphorylation in monolayer medium (Fig. 3D, E). Thus, GM-CSF was responsible for JAK2-dependent IM resistance of CML CD34^+^ cells in BM microenvironment. Recently, more evidences had proved that DNMT1 silenced tumour-suppressor gene expression by methylation of promoter DNA sequences, which played critical roles in regulation of drug resistance in cancer chemotherapy [30, 31]. In addition, GM-CSF was able to induce p15 CpG island methylation via DNMT(s) and HDAC(s) [32]. Now, we investigated whether GM-CSF promoted IM resistance through regulating DNMT1 which led to JAK2/STAT5 phosphorylation in BM microenvironment? As shown in Fig. 3D, E, GM-CSF could dose-dependently enhanced DNMT1 expression in monolayer medium. Supplementing anti-rhGM-CSF antibodies exhibited downregulation of DNMT1 expression in co-culture model in Fig. 3F, G. Furthermore, the results of ELISA showed DNMT1 activity could be enhanced significantly in GM-CSF-added monolayer medium and co-culture system. On the contrary, administration of anti-rhGM-CSF antibodies could obviously decrease DNMT1 activity in co-culture system(Fig. 3H, I).

### Baicalein overcame the protective effect of microenvironment by inhibiting JAK2/STAT5 signaling

**Fig. 4:**
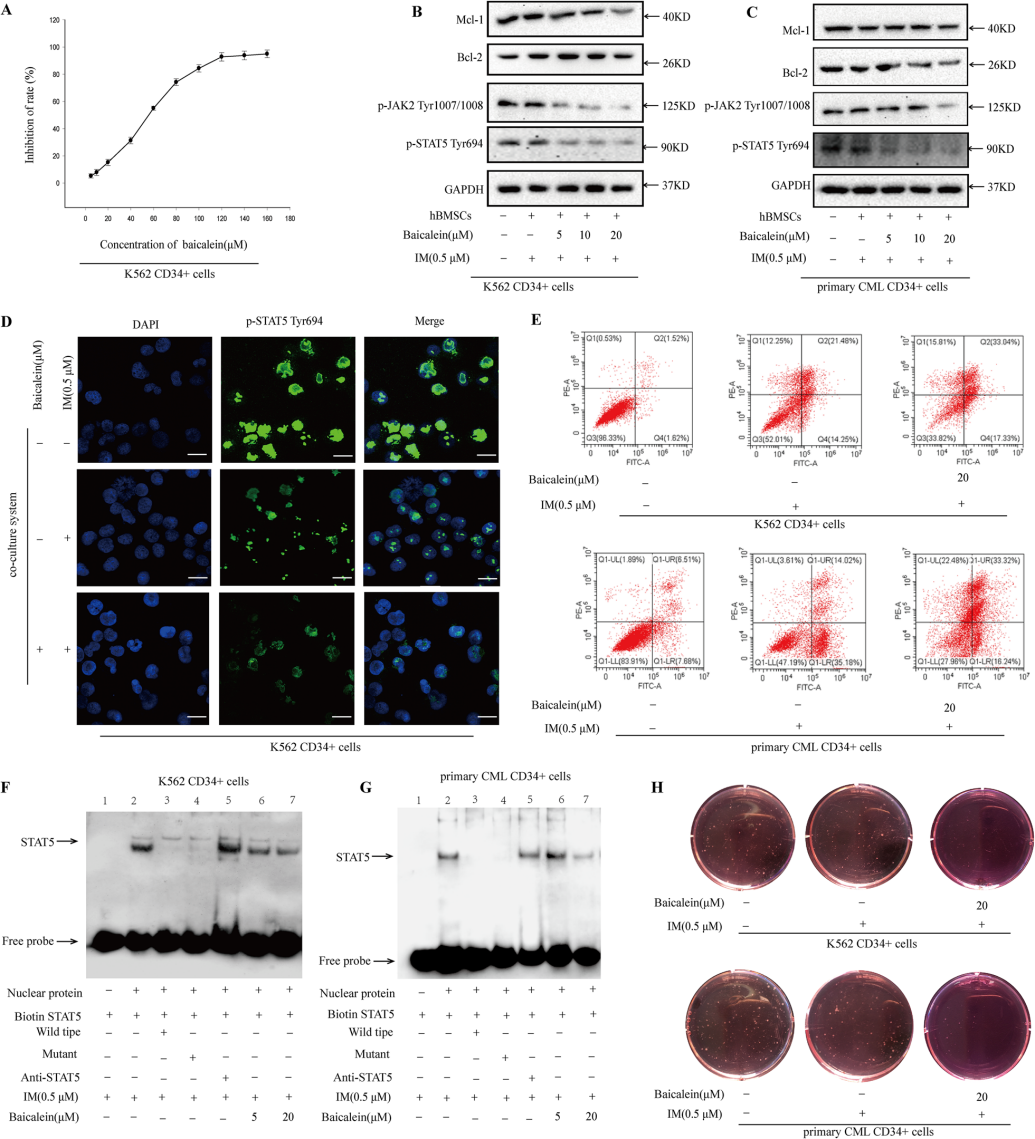
The effect of Baicalein on IM resistance of CML CD34^+^ cells within microenvironment. **A **Effects of Baicalein on proliferation of K562 CD34^+^ cells were measured by MTT analysis. The cells were treated with various concentrations of Baicalein for 36 h in the supernatant of hBMSCs. **B**, **C** K562 CD34^+^ cells and primary CML CD34^+^ cells were exposed to 0.5 μM IM within various concentrations of Baicalein for 36 h in co-culture model, respectively. JAK2/STAT5 signaling pathway was determined by Western blot in treated CML CD34^+^ cells in co-culture model. **D** Nuclear translocalization of p-STAT5^Tyr694^ (green) was acquired by confocal microscopy after staining the indicated antibody, when K562 CD34^+^ cells treated with or without 20 μM Baicalein in 0.5 μM IM-induced co-culture model. Scale bar, 10 μm. **E** K562 CD34^+^ cells and primary CD34 + CML cells were cultured with or without hBMSCs for 12 h and then treated with or without 0.5 μM IM within various concentrations of Baicalein for 36 h, respectively. Apoptosis was assessed by Annexin V-PI double staining after treatment in co-culture model. **F**, **G** K562 CD34^+^ cells and primary CML CD34^+^ cells were cultured with or without hBMSCs for 12 h and then treated with the combination of 0.5 μM IM and Baicalein for 36 h in co-culture model, respectively. STAT5 DNA binding activity was assessed by EMSA. **H** Soft-sugar-colony forming experiment was performed to ascertain Baicalein reversal effect after treatment with or without 0.5 μM IM or the combination

Base on the above results, the effects of Baicalein on JAK2/STAT5 signaling were observed in microenvironment model. To evaluate the toxicity of Baicalein, MTT was used. As indicated in Fig. 4A, the viability of K562 CD34^+^ cells was dose-dependently repressed by Baicalein. To minimize the impacts of Baicalein on proliferation of CD34^+^ cells, lower concentrations (5, 10 and 20 μM) of Baicalein were chosen in the next experiments. Treatment with Baicalein and IM for 36 h, the activation of JAK2/STAT5 signaling was obviously inhibited (Fig. 4B, C). Additionally, Baicalein significantly reduced the expression of Bcl-2 and Mcl-1 in microenvironment (Fig. 4B, C). Immunofluorescence indicated that Baicalein could decrease the higher expression of p-STAT5^Tyr694^ in nucleus(Fig. 4D). EMSA was applied to assess the effect of Baicalein on the binding activity of STAT5. As illustrated in Fig. 4F, G, STAT5 effectively bound to the biotin-labeled oligonucleotide probe as shown in lane 2. Although dealing with IM, the binding activity was still higher in BM microenvironment. However, combination of Baicalein inhibited recruitment of STAT5 to DNA as shown in lane 6 and lane 7. These data supported Baicalein could effectively suppress JAK2/STAT5 pathway of CD34^+^ cells in BM microenvironment. In addition, the combination markedly enhanced apoptosis rate of CML CD34^+^ cells in BM microenvironment (Fig. 4E). Moreover, a decrease of colony-forming capacity in BM microenvironment was also to be confirmed in combined group (Fig. 4H). Altogether, within model, Baicalein in a safe dose could reverse IM resistance through repressing proliferation of CML CD34^+^ cells via JAK2/STAT5 inactivation.

### Baicalein inhibited JAK2/STAT5 signaling by re-expressing DNMT1-mediated SHP-1

**Fig. 5:**
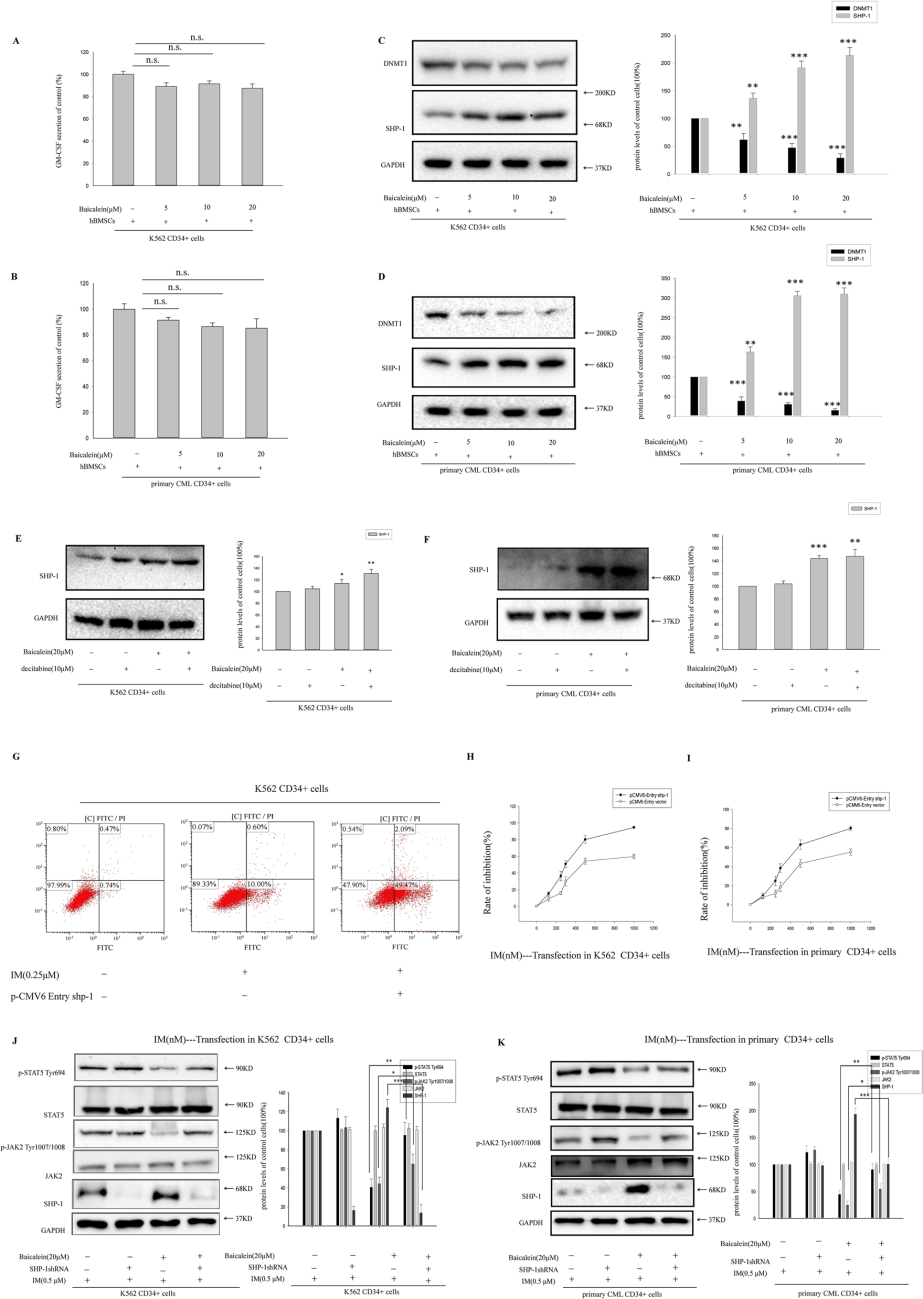
The effect of Baicalein on DNMT1-mediated SHP-1 within BM microenvironment. **A**, **B** Effects of Baicalein on GM-CSF secretion in K562 CD34^+^ cells and primary CD34^+^ CML cells were detected by ELISA assay, respectively. **C**, **D** Effects of Baicalein on DNMT1 and SHP-1 expression were determined in both CML CD34^+^ cells. **p < 0.01, ***p < 0.001 versus control group (without Baicalein treatment). **E**, **F** The expressions of SHP-1 were determined, after treatment with 10 μM DNMT1 inhibitor(decitabine) in CML CD34^+^ cells. *p < 0.05, **p < 0.01, ***p < 0.001 versus control group, Data points of 3 independent experiments are depicted. **G** pCMV6-Entry shp-1 and pCMV6-Entry vector were transfected into CML CD34^+^ cells. Subsequently, apoptosis was measured by Annexin V-PI double staining assay after treatment with IM in co-culture model. **H**, **I** After transfection, the growth inhibition effect of IM on CD34^+^ subpopulation in K562 cells or primary CML cells with or without the supernatant of hBMSCs was detected by MTT. **J**, **K** Silence of SHP-1 by SHP-1shRNA reversed the effects of Baicalein on p-JAK2^Tyr1007/1008^ and p-STAT5 ^Tyr694^ (*p < 0.05, **p < 0.01, ***p < 0.001)

To explore the repressive mechanism of Baicalein, we firstly investigated the effect of Baicalein on GM-CSF secretion in vitro. ELISA indicated that no significant changes were found in GM-CSF secretion with or without Baicalein treatment (Fig. 5A, B), suggesting that GM-CSF was not involved in the inhibitory action of Baicalein on JAK2/STAT5 signaling. Next, we examined the effect of Baicalein on DNMT1 expression. Baicalein alone markedly decreased DNMT1 in a dose-dependent manner in BM microenvironment (Fig. 5C, D).

SHP-1 negatively regulated cellular processes and involved in oncogenesis including JAK/STAT signaling [33]. The CpG methylation of SHP-1 was mediated by DNMT1 in CML [15]. As illustration in Fig. 5C, D, Baicalein alone enhanced the SHP-1 expression encoded by PTPN6 in CML CD34^+^ cells in BM microenvironment. Using 10 μm decitabine(the DNA methyltransferase inhibitor), the increasing trend of SHP-1 was consistent with Baicalein-treated cells (Fig. 5E, F). Transfection a lentiviral vector expressing SHP-1 was examined in K562 CD34^+^ cells. Overexpression of SHP-1 could increase IM induced apoptosis (Fig. 5G) and suppress cell growth(Fig. 5H, I) compared with the control vector in monolayer culture. To further understand the roles of SHP-1 in Baicalein-mediated inhibition of JAK2/STAT5 signaling, SHP-1 was inhibited by using SHP-1-targeted shRNAs. Our studies demonstrated that blocking of SHP-1 by SHP-1 shRNA (Fig. 5J, K) attenuated Baicalein-mediated inhibition of JAK2/STAT5 signaling in both CML CD34^+^ cells in co-culture model. These data suggested that Baicalein increased TKI-induced apoptosis by activating epigenetical SHP-1 negative regulator.

### DNMT1-mediated demethylation of SHP-1 was involved in the inhibition of Baicalein

**Fig. 6:**
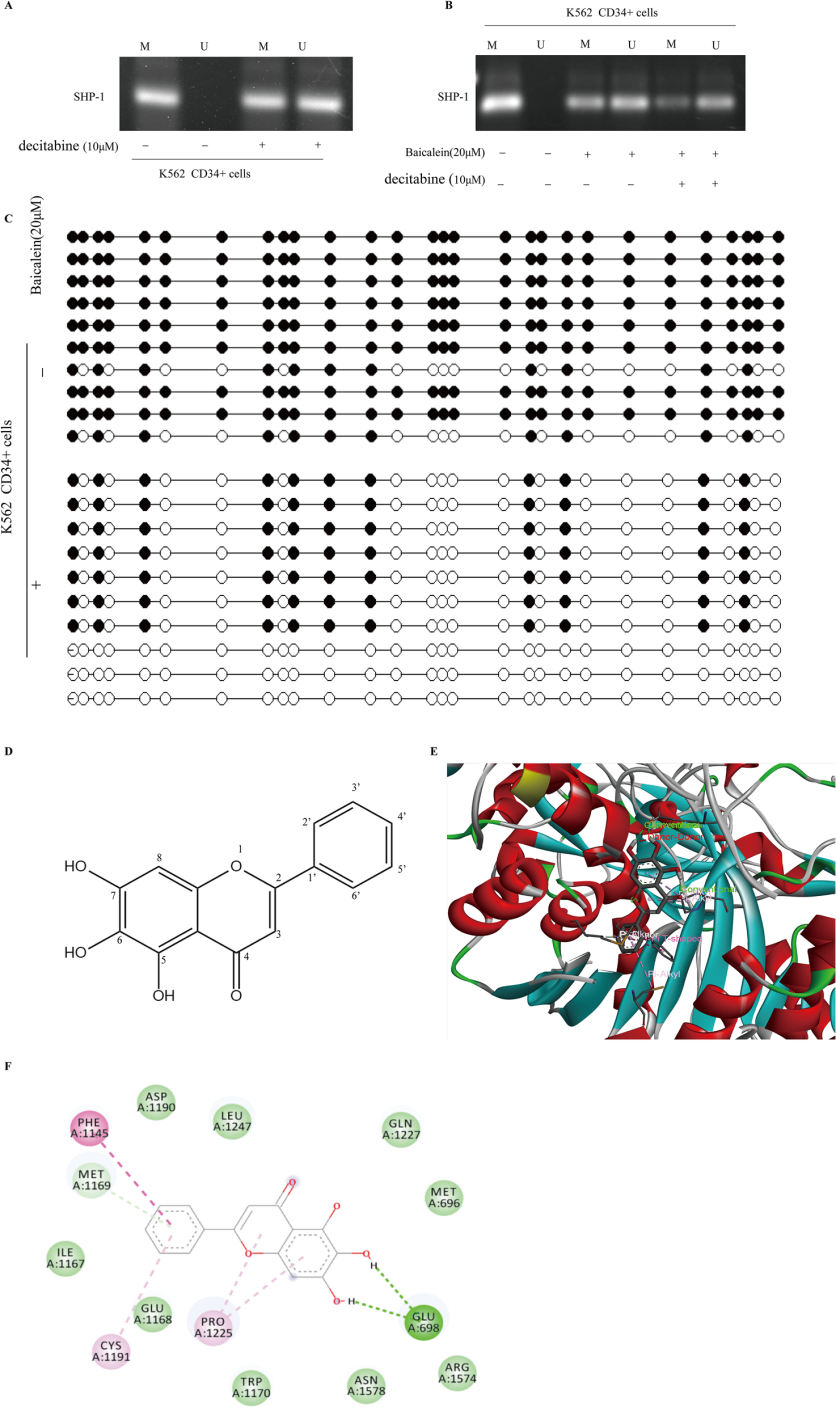
DNMT1-mediated demethylation of SHP-1 was involved in the inhibition of Baicalein. **A** The extent of SHP-1 methylation was determined by MSP using methylation‑specific primers. U, unmethylated‑specific primers; M, methylated‑specific primers. SHP-1 methylation was determined by PCR agarose gel electrophoresis after treatment with decitabine. **B** Effects of Baicalein on SHP-1 methylation were evaluated by MSP, after treatment with or without decitabine. **C** K562 CD34^+^ cells were treated with 20 μM Baicalein for 36 h in the supernatant of hBMSCs. The methylation status of SHP-1 was analyzed by BSP. Black and white dots represented methylated and unmethylated CpG dinucleotides, respectively. **D**The structure of Baicalein. **E** Docking result of the ligand Baicalein and DNMT1 was showed in 3D structure. Baicalein formed 2 hydrogen bonds (shown in green dashed lines) with DNMT1 and the -CDOCKER interaction energy was 46.5. **F** Docking result of the ligand Baicalein and DNMT1 was showed in 2D structure. Two hydrogen bonds were formed between Baicalein and DNMT1 as shown in green dashed lines. The pink dashed lines was shown as the aromatic stacking of Pi-alkyl. Viridescence was indicated as Van der Waals forces. Magenta dashed lines was shown as pi-pi stacking interaction

Base on the above results, the SHP-1 methylation status was considered in Baicalein-treated CML CD34^+^ cells. Methylated extent of the SHP-1 promoter was determined by PCR gel electrophoresis in treatment with decitabine or Baicalein. In Fig. 6A, decitabine treatment resulted in an obvious reduction of methylation of SHP-1. Consistent with the effect of decitabine treatment, an obvious reduction in the SHP-1 methylation level was observed in the Baicalein-treated cells from hypermethylation to demethylation, as shown in Fig. 6B. Meanwhile, BSP analysis furtherly showed Baicalein induced demethylation of SHP-1 at CpG sites(Fig. 6C). It suggested that Baicalein successfully induced the progressive demethylation of SHP-1, leading to re-expression of silencing SHP-1. Therefore, we proposed that Baicalein could induce SHP-1 promoter demethylation and enhance SHP-1 expression by inhibiting DNMT1 expression, resulting in inhibition of JAK/STAT signaling.

Furthermore, we speculated that DNMT1 may be a potential target of Baicalein for the treatment of CML resistance in BM microenvironment. A docking model of DNMT1 and Baicalein was evaluated by calculating the binding stability of the small-molecule ligand to the enzyme. The number of carbon atoms of Baicalein was shown in Fig. 6D. The molecular docking of hDNMT1 (PDB id: 3SWR) and Baicalein was performed by using Autodock Vina 1.5.7. Visualisation of molecular structures was used by Discovery Studio 4.5. As illustration in Fig. 6E, the results indicated that DNMT1 and Baicalein had binding pockets in 3D structures. In 2D structures of Fig. 6F, it could be seen that the benzene ring in the inhibitor Baicalein had pi-pi stacking interaction on Phe1145(shown in magenta dashed lines). The ligand C6 and C7 on the hydroxyl group had significant hydrogen bonds with the side chain of Glu698 respectively. The ligand Baicalein formed aromatic stacking of Pi-alkyl at the Cys1191 and Pro1225 side chain (shown in pink dashed lines). Van der Waals forces were built between small molecule ligand and many amino acids (shown in viridescence). The -CDOCKER interaction energy was 46.5. Thus, these findings further supported Baicalein might be a small-molecule inhibitor targeting DNMT1 to reverse IM resistance in CML patients.

### Baicalein enhanced the therapeutic effect of IM on leukemia development in vivo

**Fig. 7:**
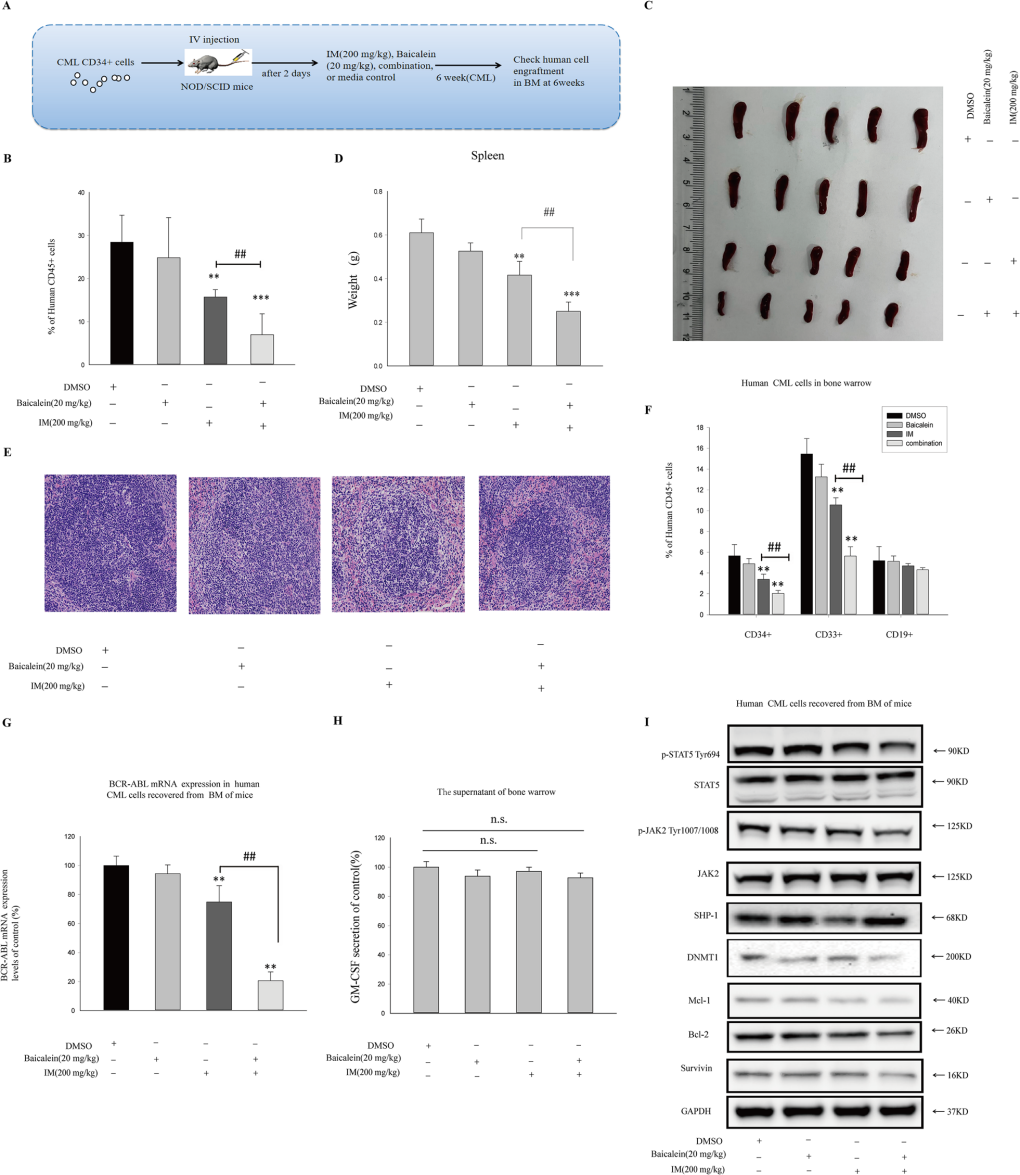
The effect of combination of IM and Baicalein on the leukemogenic activity engrafted with CML CD34^+^ cells in vivo. **A** K562 CD34^+^ cells (2 × 10^6^ cells per mouse) were transplanted into NOD/SCID mice, and then engraftments were randomly divided into four groups respectively. The animals engrafted with CD34^+^ cells were treated with or without IM (200 mg/kg), combination with or without Baicalein (20 mg/kg) (four groups, 5 mice/per group: media control(DMSO); Baicalein; IM; Baicalein + IM). Mice were performed with death after 6 weeks, and bone marrow contents were obtained. **B** Flow cytometry graphs showed that the levels of human CML CD45^+^ cells were regenerated in the BM of mice engrafted with K562 CD34 + cells treated with different drugs at 6 weeks. **C** Images of spleen were obtained from mice in each group. (**D**) Weight of spleen were examined and analyzed, **p < 0.01, ***p < 0.001 versus control group (DMSO); ^##^*p* < 0.01 versus IM group. **E** The serial sections of spleens were examined for H&E staining. **F** The levels of human CML CD34^+^, CD33^+^, and CD19^+^ cells regenerated in the BM of mice engrafted with K562 CD34^+^ cells treated with different drugs were measured at 6 weeks. **G** The expressions of BCR-ABL mRNA obtained from selected CD45 + cells in BM were measured by qRT-PCR and normalized to GAPDH mRNA levels. **H** GM-CSF secretion in the supernatant of BM was evaluated. **I** The effect of Baicalein on JAK2/STAT5 signaling pathway was observed by Western blot from BM CML cells of mice. All data from independent experiments are presented as means ± SD. Significance values: **p < 0.01,***p < 0.001 versus control group; ^##^*p* < 0.01 versus IM group

The above data indicated that Baicalein could effectively reverse drug resistance to IM in CML CD34^+^ cells in BM microenvironment. To verify the therapeutic potential of Baicalein, the inhibitory effects of IM and Baicalein, individual or combined, were compared against CML CD34^+^ cells in engrafted NOD/SCID mice (Fig. 7A). K562 CD34^+^ cells were injected into irradiated NOD/SCID mice by intravenous respectively, and then engraftments treated with IM (200 mg/kg), Baicalein(20 mg/kg), or the combination was evaluated. 6 weeks later, reconstitution of human CD45^+^ hemopoiesis by CML CD34^+^ cells was detected. The data indicated that CD45^+^ hemopoiesis from mice BM was reduced in IM group than in DMSO group(Fig. 7B). However, the combination induced an obvious decrease of CD45^+^ hemopoiesis in NOD/SCID mice engrafted with CML CD34^+^ cells (Fig. 7B). Spleens of engrafted group showed a significant congestion and edema, large size and loaded-weight(Fig. 7C). Spleens in combination group generally recovered to size and weight of normal spleens, showing the markedly reduced phenomenon in congestion and edema compared with IM or Baicalein, individual(Fig. 7C, D). As shown in Fig. 7E, HE staining of spleens in leukemia induced group exhibited infiltration of numerous myeloid cells and immature megakaryocytes, as well as infiltration of neoplastic cells with large nuclei. Conversely, treatment in combination relieved these symptoms of spleens significantly. It is worth mentioned that both the expression of CD34^+^ and CD33^+^ cells in myeloid engraftment were also markedly decreased by the combination(Fig. 7F). Meanwhile, qRT-PCR analysis demonstrated that the combination downregulated BCR-ABL mRNA during residual cells from BM (Fig. 7G). To further confirm the potential mechanism of Baicalein-mediated antitumor activity in vivo, we analyzed the expression of DNMT1, SHP-1, p-JAK2^Tyr1007/1008^, p-STAT5^Tyr694^, as well as secretion of GM-CSF. Treatment with Baicalein had no significant effects on GM-CSF secretion (Fig. 7H), but markedly down-regulated the expression of DNMT1, p-JAK2^Tyr1007/1008^, p-STAT5^Tyr694^, and its downstream signaling target in Fig. 7I.

Taken together, these data indicated that Baicalein induced DNMT1-dependent demethylation of the SHP-1 promoter region, and subsequently activated SHP-1 expression, which resulted in an inhibition of JAK2/STAT5 signaling in resistant CML CD34^+^ cells. Thus, DNMT1 targeting was essential in Baicalein-mediated reversal effect on resistance in BM microenvironment.

## Discussion

Although effective inhibitions of BCR-ABL tyrosine kinase activity, minimal residual cells still existed in CML patients after receiving TKI treatment [34]. Therefore, it is necessary to explore novel therapeutic strategies for reversing the acquired resistance of IM. Baicalein has been reported to reverse drug-resistance in many kinds of carcinoma cells. This studies were to assess whether Baicalein could reverse IM resistance in CML minimal residual cells, and explore the possible mechanisms. Our results showed that BM microenvironment-derived hBMSCs contributed to CML CD34^+^ cells resistance to IM via activating JAK2/STAT5 signaling, and Baicalein provided a promising strategy for reversing it by enhancing DNMT1-dependent demethylation of the SHP-1.

Our observation showed that JAK2/STAT5 signaling played a vital role in protecting CML CD34^+^ cells from TKI-inducing death in BM microenvironment. Additional drivers and prooncogenic pathways could induce the activation of JAK2/STAT5 signalling in CML patient [35]. Therefore, we analyzed the relationship between cytokines and JAK2/STAT5 activity. The results showed that GM-CSF conferred CML CD34^+^ cells resistance to IM. Within different conditions, GM-CSF-mediated JAK2/STAT5 activation circumvented the dependence on BCR-ABL signaling to support survival. Further researches revealed GM-CSF promoted IM resistance through regulating DNMT1, leading to JAK2/STAT5 phosphorylation in BM microenvironment. Base on the data, we concluded that GM-CSF-initiated JAK2/STAT5 signaling activation via DNMT1 could be functionally remedy for BCR-ABL-mediated STAT5 activation, when BCR-ABL kinase activity was impeded by IM. Hence, it became essential survival signals in protecting CML CD34^+^ cells from apoptosis in BM microenvironment.

The suppression of JAK2/STAT5 signaling was an effective path to overcome resistance to TKIs. However, JAK2 inhibitors were still fairly controversial due to its side effects. The increasing evidence showed Baicalein was effective antileukemic properties and low-toxicities. Hence, we investigated the effect of Baicalein on activity of JAK2/STAT5 signaling. We found that Baicalein in a safe dose enhanced IM-induced apoptosis via inhibiting JAK2/STAT5 pathway in vivo or in vitro. To further understand the detailed mechanism of Baicalein-mediated inhibition, we first considered the effect of Baicalein on GM-CSF secretion. However, ELISA showed that there was no association between the inhibition effect of Baicalein and GM-CSF secretion in BM or hBMSCs. SHP-1 negatively regulated cell cycle, JAK/STAT pathways, as well as inflammatory in cancer progression [36]. Minoo et al. reported SHP-1 functioned as a negative regulator of cytokine-induced STAT5 activation [37]. DNMT1 was reported to be essential for epigenetic reprogramming of SHP-1 in persistence of CML LSCs [38].

So, we next separately examined the effect of Baicalein on the expression of DNMT1 and SHP-1 in BM microenvironment. Our evidence supported that Baicalein significantly reduced DNMT1 at protein level in a dose-dependent manner. Meanwhile, Baicalein-treated cells showed a marked increase in the SHP-1 level in CML CD34^+^ cells. Methylation in the promoter regions of SHP-1 gene played an essential role in the formation of malignant haematological diseases [39]. DNMTl can participate in gene methylation [40]. We then considered whether Baicalein-mediated apoptosis was associated with reduction of SHP-1 methylation. After adminstration of 10 μm decitabine, the increasing trend of SHP-1 was detected both in CML CD34^+^ cells. Meanwhile, overexpression of SHP-1 could increase IM-induced apoptosis and suppress cell growth compared with the control vector in monolayer culture. Knockdown of SHP-1 by SHP-1 shRNA attenuated Baicalein-mediated inhibition of JAK2/STAT5 signaling in both CML CD34^+^ cells in co-culture. These results indicated an increase of TKI-induced apoptosis was related with SHP-1 re-expression in Baicalein treatment in CML CD34^+^ cells. Consistent with the effects of decitabine treatment, MSP results further showed Baicalein-treated cells had a significant decrease in the methylation level of SHP-1. It suggested that Baicalein overcame CML CD34^+^ cells resistance to IM by successfully inhibiting DNMTl and inducing the progressive demethylation of SHP-1 in BM microenvironment. The auto-inhibitory function of SHP-1 is supported by various intermolecular interactions between N-SH2 and PTP catalytic domain [41]. Baicalein might be able to switch SHP-1 structure from autoinhibitory (closed) to active (open) via demethylation of SHP-1. Base on the findings, it is therefore reasonable to speculate that DNMT1 may be a potential target of Baicalein for CML resistance therapeusis in BM microenvironment. Furthermore, the molecular docking of hDNMT1 (PDB:3SWR) and Baicalein was performed by computer simulation. The results showed DNMT1 and Baicalein had binding pockets in 3D structures. The ligand C6 and C7 on the hydroxyl group had significant hydrogen bonds with the side chain of Glu698 respectively. Aromatic stacking of Pi-alkyl, pi–pi stacking interaction,Van der Waals forces were formed to ensure more stability in molecular docking. The binding between DNMT1 and Baicalein were stronger at lower binding energy. These findings further supported Baicalein might be a small-molecule inhibitor targeting DNMT1 to reverse IM resistance in CML patients.

## Conclusions

**Fig. 8:**
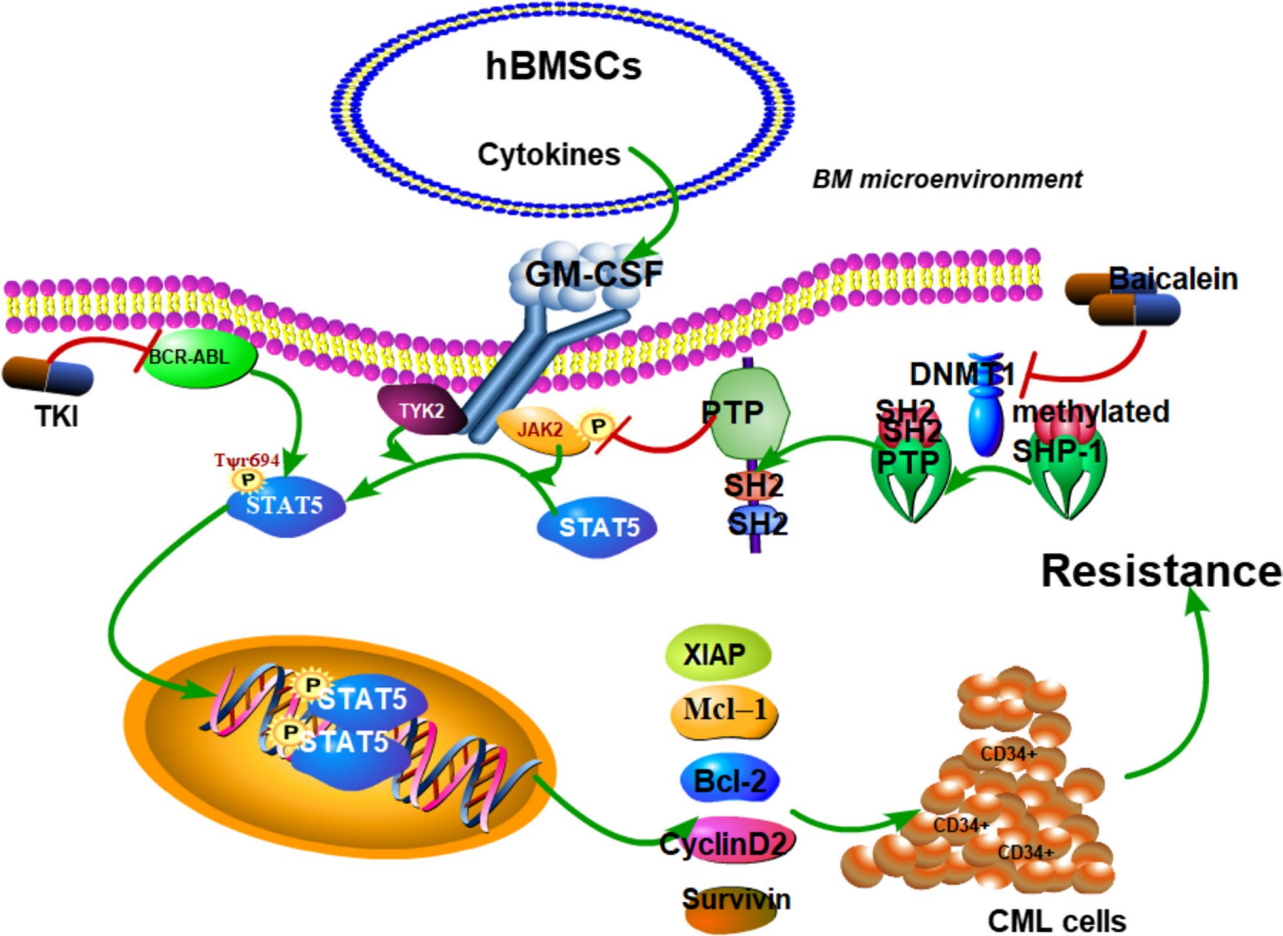
Schematic diagram illustrated that Baicalein overcame IM resistance by reducing DNMT1-mediated methylation of SHP-1 and inducing an inhibition of JAK2/STAT5 signaling in CML CD34^+^ cells

As shown in Fig. 8, it presented a reasonable mechanism that Baicalein enhanced IM inhibition through suppressing JAK2/STAT5 pathway against CML CD34^+^ cells in vivo and in vitro. The mechanism of Baicalein on improving the sensitivity of CD34^+^ cells to IM might be correlated with SHP-1 demethylation by inhibition of DNMT1 expression. Further, the molecular docking indicated that Baicalein might be a small-molecule ligand of DNMT1 by hydrogen bonds and hydrophobic interactions. Therefore, identification of Baicalein could be a potent DNMT1 inhibitor to repress JAK2/STAT5 survival pathway in CML CD34^+^ cells. This might emerge an alternative strategy by targeting DNMT1 in the treatment of CML resistant patients.

## Data Availability

All data generated or analysed during this studies was included in this published article and its supplementary information files.

## Abbreviations

CML: Chronic myeloid leukemia
IM: Imatinib
BM: Bone marrow
SFM-DR: Soluble factor-mediated drug resistance
TKIs: TK inhibitors
DNMT1: DNA-methyltransferase 1
SHP-1: Src homology region 2 domain-containing phosphatase-1
JAK2: Janus kinase 2
STAT5: Signal transducer and activator of transcription 5
hBMSCs: Human bonemesenchymal stem cells
MSP: Methylation‑specific PCR
BSP: Bisulfite sequencing PCR
